# Schuhmann’s Question Compend

**Published:** 1914-11-15

**Authors:** 


SCHUHMANN'S QUESTION COMPEND. This is a small book published by Dr. H. H. Schuhmann, Marshall Field Annex Building, Chicago, giving in substance the course of study by the Chicago Dental Research Club last year under the direction of Dr. Edward H. Baker. Dr. Schuhmann has made up a complete list of questions with brief answers from notes taken during the course. The subjects covered are; Urine, Blood, Cell Life, Immunity, Food, Chemistry of Metabolism, Enzymes, Fats and Ethers, Diffusion, Dialysis and Osmosis, Syphilis, Nephritis.

